# Cholesterol-enriched membrane micro-domaindeficiency induces doxorubicin resistancevia promoting autophagy in breast cancer

**DOI:** 10.1016/j.omto.2021.10.005

**Published:** 2021-10-20

**Authors:** Yin Shi, Zu Ye, Guang Lu, Naidi Yang, Jianbin Zhang, Liming Wang, Jianzhou Cui, Miguel A. del Pozo, Yihua Wu, Dajing Xia, Han-Ming Shen

**Affiliations:** 1 Department of Immunology, Zhejiang University School of Medicine, Hangzhou 310058, China; 2 Department of Physiology, Yong Loo Lin School of Medicine, National University of Singapore 119077, Singapore; 3 Department of Molecular and Cellular Oncology, The University of Texas MD Anderson Cancer Center, Houston 77030, USA; 4 Key Laboratory of Flexible Electronics (KLOFE) & Institute of Advanced Materials (IAM), Nanjing Tech University, Nanjing, Jiangsu Province 211800, China; 5 School of Biomedical Science, Hunan University, Changsha, Hunan, China; 6 Integrin Signaling Laboratory, Vascular Biology and Inflammation Department, Centro Nacional de Investigaciones Cardiovasculares, Madrid 28029, Spain; 7 Department of Toxicology of School of Public Health, and Department of Gynecologic Oncology of Women's Hospital, Zhejiang University School of Medicine, Hangzhou 310058, China; 8 Faculty of Health Sciences, University of Macau, Macau SAR 999078, China

**Keywords:** autophagy, breast cancer, doxorubicin resistance, cholesterol-enriched membrane micro-domains, CAV1, VAMP3

## Abstract

Drug resistance has become one of the largest challenges for cancer chemotherapies. Under certain conditions, cancer cells hijack autophagy to cope with therapeutic stress, which largely undermines the chemo-therapeutic efficacy. Currently, biomarkers indicative of autophagy-derived drug resistance remain largely inclusive. Here, we report a novel role of lipid rafts/cholesterol-enriched membrane micro-domains (CEMMs) in autophagosome biogenesis and doxorubicin resistance in breast tumors. We showed that CEMMs are required for the interaction of VAMP3 with syntaxin 6 (STX6, a cholesterol-binding SNARE protein). Upon disruption of CEMM, VAMP3 is released from STX6, resulting in the trafficking of ATG16L1-containing vesicles to recycling endosomes and subsequent autophagosome biogenesis. Furthermore, we found that CEMM marker CAV1 is decreased in breast cancer patients and that the CEMM deficiency-induced autophagy is related to doxorubicin resistance, which is overcome by autophagy inhibition. Taken together, we propose a novel model whereby CEMMs in recycling endosomes support the VAMP3 and STX6 interaction and function as barriers to limit the activity of VAMP3 in autophagic vesicle fusion, thus CEMM deficiency promotes autophagosome biogenesis and doxorubicin resistance in breast tumors.

## Graphical abstract

**Figure:**
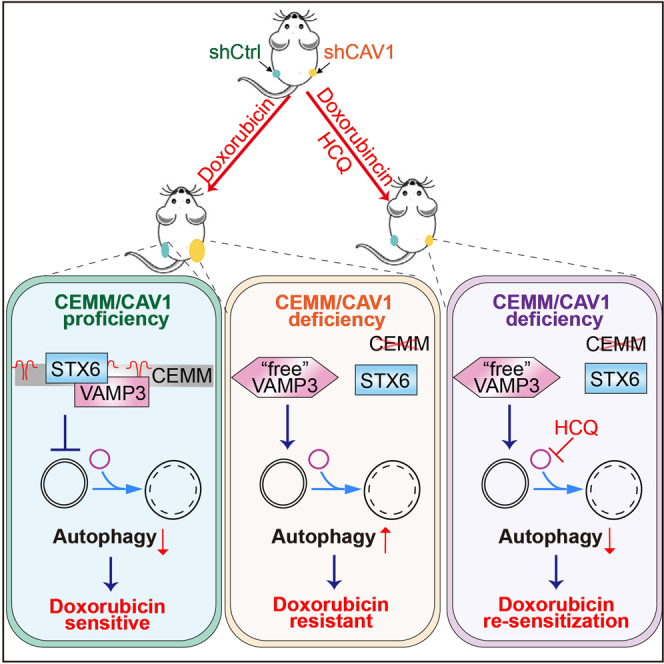

## Introduction

Macroautophagy (here referred to as autophagy) is an evolutionarily conserved “self-eating” process that results in degradation of long-lived proteins and organelles via the lysosomal pathway, which is essential for the maintenance of cellular homeostasis.^1^ Although the role of autophagy in different stages of different tumors remains elusive, increasing evidence demonstrates that, in the context of cellular responses to cancer therapy, autophagy is instrumental in drug resistance.^2^, ^3^, ^4^ The autophagy process includes two consecutive stages: (1) formation of phagophores/isolation membranes, and autophagosome (early stage), and (2) fusion of autophagosomes with lysosomes, and subsequent lysosomal degradation (late stage).^5^ At present, the mechanisms controlling autophagosome biogenesis have been extensively studied and various autophagy-related (ATG) proteins are involved in this process.^6^ These ATG proteins form a series of complexes, such as the unc-51 like autophagy activating kinase 1 (ULK1) complex (consisting of ULK1, ATG13, ATG101, and RB1 inducible coiled-coil 1 [RB1CC1]) and the phosphatidylinositol 3-kinase catalytic subunit type 3 (PIK3C3)-Beclin 1 (BECN1) complex (mainly consisting of PIK3C3, BECN1, ATG14L, VPS15, and p150), leading to the generation of PtdIns3P.^7^^,^^8^ Subsequently, the PtdIns3P effector WD repeat domain, phosphoinositide interacting 2 (WIPI2) further recruits the ATG12-ATG5-ATG16L1 complex, which functions as an E3 to facilitate the lipidation of microtubule associated protein 1 light chain 3 (LC3) and ultimately the maturation of autophagosomes.^9^, ^10^, ^11^ In addition to the key ATGs mentioned above, autophagosome formation requires membrane fusion driven by SNAREs (SNAP [soluble NSF attachment protein] receptor), a family of proteins known to mediate membrane/vesicle fusion events.^12^, ^13^, ^14^, ^15^, ^16^, ^17^ Recent studies suggest that SNAREs are not solely involved in autophagosome-lysosome fusion. For example, the v-SNARE protein vesicle-associated membrane protein 3 (VAMP3) is reported to mediate heterotypic fusions between ATG9- and ATG16L1-containing vesicles in recycling endosomes, which correlates well with the autophagosome formation process.^12^ Another v-SNARE protein, syntaxin 17 (STX17), which is well known as a key player in autophagosome-lysosome fusion, is reported to promote Atg14L accumulation at ER-mitochondria contact sites, leading to omegasome formation on the ER.^15^^,^^18^ These findings together indicate that SNAREs also function in autophagosome biogenesis.

Cholesterol-rich domains in the cellular membrane system named “lipid rafts” or cholesterol-enriched membrane micro-domains (CEMMs) are liquid-ordered dynamic micro-domains composed of a characteristic structural composition (sphingolipids, cholesterol, and proteins) in membrane systems, including Golgi, ER, mitochondria, recycling endosomes, and endosomes/lysosomes.^19^, ^20^, ^21^, ^22^ In recent years, CEMMs have been reported to play a crucial role in various cellular processes, including cellular signaling transduction, cytoskeletal organization, and membrane trafficking.^23^, ^24^, ^25^, ^26^ At present, the function of CEMMs in autophagy remains not fully understood. Although there are some reports showing that CEMMs promote initiation of autophagy,^27^, ^28^, ^29^, ^30^, ^31^ disruption of CEMMs is known to induce autophagy in both mice models and in various tissues and cancer cells, such as lung epithelial cells, vascular endothelium, osteosarcoma, and breast and liver cancer cells.^32^, ^33^, ^34^, ^35^, ^36^, ^37^, ^38^, ^39^, ^40^ For instance, Caveolin 1 (CAV1), a scaffolding protein critical for CEMM structure and cholesterol homeostasis, has been reported to interact with the ATG12-ATG5 conjugation system and suppress autophagy in lung epithelial cells and aortic endothelium.^34^^,^^41^ Consistently, our previous study showed that CAV1 acts with CEMMs to inhibit autophagy via negative regulation of the lysosomal function.^32^ It appears that CEMMs modulate autophagy distinctively under different contexts. In fact, modulation of autophagy via targeting CEMMs drives more and more attention in the fields of anti-cancer strategies, neurodegenerative diseases, and anti-COVID-19 therapy.^5^^,^^32^^,^^36^^,^^40^^,^^42^, ^43^, ^44^, ^45^ Thus, how exactly CEMMs regulate autophagy remains an important question.

In this study, we provide evidence demonstrating that CEMM disruption decreases the interaction between a cholesterol-binding SNARE protein STX6 and an autophagy-related SNARE protein VAMP3 at recycling endosomes. Subsequently, the released VAMP3 in recycling endosomes promotes the trafficking of ATG16L1-containing vesicles to recycling endosomes and subsequent autophagosome biogenesis. Moreover, we show that autophagy resulting from CEMM disruption is involved in doxorubicin (Doxo) resistance in breast cancer cells with low CAV1 expression, while suppression of autophagy by hydroxychloroquine (HCQ) overcomes the drug resistance. Our findings thus provide novel insights into the molecular mechanisms controlling autophagosome biogenesis via SNARE protein in recycling endosomes under the regulation of CEMMs, and the potential clinical target for breast cancer patients with reduced CAV1 expression who develop Doxo resistance.

## Results

### CEMM disruption promotes autophagic flux

To examine the function of CEMM in autophagy, we manipulated cellular cholesterol levels by methyl-β-cyclodextrin (MBCD) depletion or a genetic approach using *Cav1*-KO, the scaffolding protein that maintains the structure of CEMM.^46^, ^47^, ^48^

After 1 h of MBCD pre-treatment, cholesterol level was dose dependently decreased in total cell lysates and maintained for at least 5 h (Figures S1A and S1B). Cholesterol replenishment completely recovered the cholesterol level (Figure S1C).^49^ We also confirmed a significant decrease of cholesterol and CEMMs in the *Cav1*-KO cells (Figure S1D). Then we utilized Filipin staining, cholera toxin subunit B (CTxB) staining, and mCherry-D4H to label and measure cellular CEMM levels.^50^, ^51^, ^52^ These CEMM markers could be significantly reduced by MBCD (Figures S1E–S1J) or *Cav1*-KO (Figures S1K–S1N), which could be fully recovered upon cholesterol replenishment. Together, these data suggest that MBCD and *Cav1*-KO can efficiently reduce cholesterol level and inhibit CEMM function.

**Figure 1:**
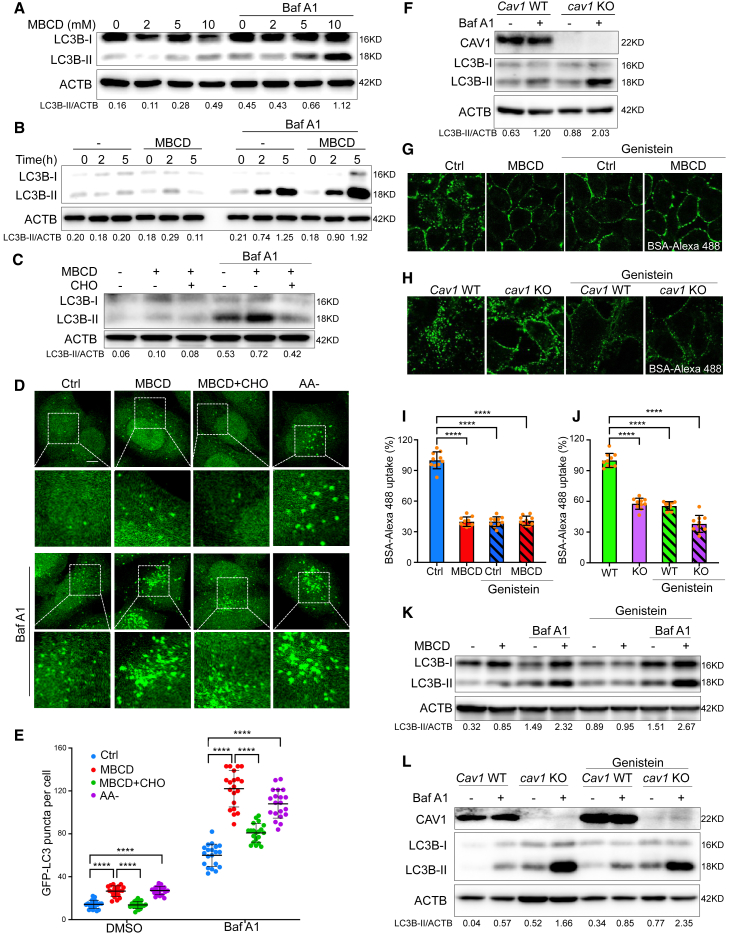
CEMM disruption promotes autophagic flux (A) HeLa cells were pre-treated with MBCD at the indicated concentration for 1 h, then incubated in the presence or absence of bafilomycin A1 (Baf A1, 100 nM) for 2 h. Cell lysates were collected and subjected to western blots for the indicated markers. (B) HeLa cells were pre-treated with or without MBCD (5 mM) for 1 h, then incubated in the presence or absence of Baf A1 (100 nM) for the indicated times. Cell lysates were collected and subjected to western blots for the indicated markers. (C) HeLa cells were pre-treated with or without MBCD (5 mM) for 1 h, then incubated in the presence or absence of cholesterol (CHO, 30 μg/mL) or Baf A1 (100 nM) as indicated for 2 h. Cell lysates were collected and subjected to western blots for the indicated markers. (D) HeLa cells with stable expression of GFP-LC3B were pre-treated with or without MBCD (5 mM) for 1 h, then incubated in the presence or absence of amino acid-deficient DMEM medium (AA–), CHO (30 μg/mL), or Baf A1 (100 nM) as indicated. The cells were observed under a confocal microscope (×600). Scale bars, 5 μm. (E) The number of GFP-LC3B puncta observed in (D) are presented as means ± SD. Statistical significance was evaluated using a two-tailed Student's t test. ∗∗∗∗p < 0.0001. (F) *Cav1* WT and c*av1* KO MEFs were treated with or without Baf A1 (100 nM) for 2 h. The total cell lysates were then immunoblotted with the indicated markers. (G) In the presence or absence of genistein (200 μM), HeLa cells were pre-treated with MBCD (5 mM, 1 h) and then incubated with BSA-Alexa 488 (50 μg/mL). (H) In the presence or absence of genistein (200 μM), *Cav1* WT and *cav1* KO MEFs were incubated with BSA-Alexa 488 (50 μg/mL). Cells were observed under a confocal microscope. (I) The intracellular uptake of BSA-Alexa 488 (the fluorescence signals inside the cytoplasm) in (G) were analyzed using ImageJ (normalized to control [Ctrl] cells). ∗∗∗∗p < 0.0001. (J) The intracellular uptake of BSA-Alexa 488 (the fluorescence signals inside the cytoplasm) in (H) were analyzed using ImageJ (normalized to WT cells). Statistical significance was evaluated with a two-tailed Student's t test. ∗∗∗∗p < 0.0001. (K) HeLa cells were pre-treated with or without MBCD (5 mM) for 1 h, then incubated in the presence or absence of genistein (200 μM) or Baf A1 (100 nM) as indicated for 2 h. (L) *Cav1* WT and *cav1* KO MEFs were treated in the presence or absence of genistein (200 μM) or Baf A1 (100 nM) as indicated for 2 h. The total cell lysates were then immunoblotted with the indicated markers.

Next, we examined autophagic flux after CEMM disruption. The changes of LC3B lipidation after MBCD pre-treatment significantly increased time and dose dependently under both normal or bafilomycin A1 (Baf A1, lysosome inhibitor) conditions (Figures 1A and 1B). Such an increase was attenuated after cholesterol replenishment (Figure 1C). The changes of GFP-LC3 puncta after cholesterol depletion or replenishment showed a similar pattern (Figures 1D and 1E). By using *Atg5* Tet-off inducible MEFs, we confirmed that the LC3B-II lipidation and GFP-LC3 puncta formation induced by CEMM disruption was Atg5 dependent (Figures S2A–S2C). Consistently, the genetic depletion of *Cav1* also significantly enhanced autophagy flux (Figure 1F).

MBCD treatment or CAV1 deficiency is known to inhibit caveolae-mediated endocytosis.^53^ To confirm that CEMM disruption-induced autophagic flux is not caused by deficiency of nutrients resulting from reduced endocytosis, we compared the autophagic flux induced by CEMM disruption with or without genistein, a caveolae-mediated endocytosis inhibitor.^54^^,^^55^ Inhibition of endocytosis after genistein was confirmed by the dramatic decrease of intracellular uptake of BSA-Alexa 488, a marker used for caveolae-mediated endocytosis (Figures 1G–1J).^56^ Notably, CEMM disruption via MBCD or CAV1 deficiency did not further reduce the intracellular uptake of BSA after genistein treatment, indicating that there was no additional inhibition of cell endocytosis. We found that, after genistein treatment, MBCD alone did not significantly enhance the amount of LC3-II (0.89 versus 0.95) (Figure 1K). This observation could be explained by the fact that genistein itself can enhance lysosome function, which may lead to increasing LC3-II degradation via the autophagy-lysosome pathway.^57^^,^^58^ So, we examined autophagic flux after lysosomal inhibition by Baf A1. We were still able to detect a significant increase of LC-3 II levels after MBCD treatment or *Cav1* KO in the presence of genistein (Figures 1K and 1L). The results indicate that CEMM disruption by MBCD or *Cav1* KO was still able to induce autophagic flux after genistein treatment at a comparable level as under normal conditions (Figures 1K and 1L). Therefore, we confirmed that the nutrient deficiency caused by inhibition of endocytosis was not the major cause for autophagic flux after CEMM disruption.

### CEMM disruption promotes autophagosome biogenesis

**Figure 2:**
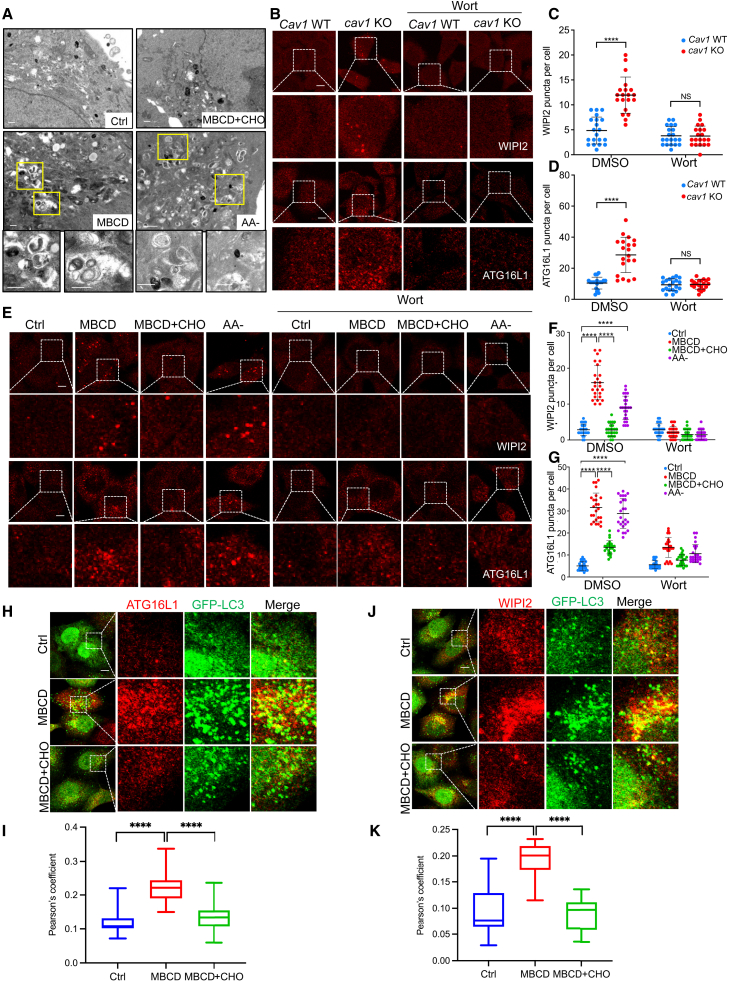
CEMM disruption promotes autophagosome biogenesis (A) Electron micrographs of HeLa cells treated as the (1) Ctrl; (2) MBCD 5mM, pre-treated 1 h, then incubated for 2 h; (3) MBCD pre-treated as described in (2), then the cells were incubated with CHO (30 μg/mL) for 2 h; (4) cells were treated in AA for 2 h as positive control. Scale bars, 0.5 μm. (B) *Cav1* WT and c*av1* KO MEFs were treated with or without wortmannin (Wort, 100 nM) for 2 h. Cells were immunostained by ATG16L1 or WIPI2, and observed under confocal microscope (×600). Scale bars, 5 μm. (C) The number of WIPI2 puncta observed in (B) are presented as means ± SD. ∗∗∗∗p < 0.0001; NS > 0.05. (D) The number of ATG16L1 puncta observed in (B) are presented as means ± SD. ∗∗∗∗p < 0.0001; NS > 0.05. (E) HeLa cells were pre-treated with or without MBCD (5 mM) for 1 h, then incubated in the presence or absence of CHO (30 μg/mL) or Wort (100 nM) as indicated for 2 h. Cells were immunostained by ATG16L1 or WIPI2, and observed under a confocal microscope (×600). Scale bars, 5 μm. (F) The number of WIPI2 puncta observed in (E) are presented as means ± SD. ∗∗∗∗p < 0.0001. (G) The number of ATG16L1 puncta observed in (E) are presented as means ± SD. ∗∗∗∗p < 0.0001. (H) HeLa cells with stable expression of GFP-LC3B were pre-treated with or without MBCD (5 mM) for 1 h, then incubated in the presence or absence of CHO (30 μg/mL) for 2 h, meanwhile Baf A1 (100 nM) was added to all the treatments to induce enough accumulation of autophagic vesicles for observing colocalization between ATG16L1 and GFP-LC3. All the cells were immunostained by ATG16L1. Scale bars, 5 μm. (I) The Pearson correlation coefficient of GFP-LC3B puncta with ATG16L1 from the experiment described in (H). ∗∗∗∗p < 0.0001. (J) HeLa cells with stable expression of GFP-LC3B were treated as described in (H), then immunostained by WIPI2. Scale bars, 5 μm. (K) The Pearson correlation coefficient of GFP-LC3B puncta with WIPI2 from the experiment described in (J). ∗∗∗∗p < 0.0001.

Given that CEMM disruption by MBCD or CAV1 deficiency can further enhance LC3 lipidation after lysosome inhibition, we hypothesized that CEMM disruption may promote autophagosome biogenesis. To address this, we examined autophagosome-like structures through electron microscopy. As expected, we found a significant increase of double-membrane autophagosome structures after MBCD treatment, similar to the positive control with amino acid starvation (AA–) treatment, while cholesterol replenishment totally reversed this phenomena (Figure 2A). Two nascent autophagosome markers, ATG16L1 and WIPI2, also could be significantly increased by MBCD or *Cav*1 deficiency and reduced by cholesterol replenishment (Figures 2B–2G). In addition, the increases of ATG16L1 and WIPI2 induced by CEMM disruption were sensitive to wortmannin (Wort), an inhibitor of PIK3C3 that is a well-established inhibitor of autophagosome formation (Figures 2B–2G), suggesting that this increase was due to induction of autophagy. The ATG16L1- or WIPI2-positive puncta showed a significant increased colocalization with GFP-LC3 puncta after MBCD treatment, indicating that the LC3 recruitment to the autophagosome was also promoted after CEMM disruption (Figures 2H–2K). Together, results from this section confirm the inductive effect of CEMM disruption on autophagosome biogenesis.

### CEMM disruption releases VAMP3 from CEMMs at the recycling endosomal membrane

CEMMs are reported to accumulate on the membrane of recycling endosomes in kidney cells.^59^ In addition, recycling endosomes are known to be the place where the ATG16L1-positive membranes coalesce and then promote autophagosome biogenesis.^12^ Therefore, we examined the distribution of CEMMs on recycling endosomes by using transferrin receptor (TFRC) as a marker for recycling endosomes.^60^ MBCD disrupts CEMMs and thus reduces the colocalization between Filipin and the TFRC, while cholesterol replenishment recovered this colocalization (Figures S3A and S3B). Thus, our results are consistent with an earlier report showing the enrichment of CEMMs in recycling endosomes.^59^ In addition, disruption of CEMMs induced the colocalization between TFRC- and ATG16L1-positive vesicles, a process reversed by cholesterol replenishment (Figures S3C and S3D). The GFP-LC3B puncta are partially co-localized with TFRC after CEMM disruption and reversed by cholesterol replenishment (Figures S3E and S3F). These data thus indicate that disruption of CEMMs promotes the coalescence of ATG16L1-positive vesicles in recycling endosomes and the subsequent formation of autophagosomes.

It has been established that the v-SNARE protein VAMP3 is critical for the ATG16L1-positive membranes to meet with other autophagosome membrane sources in recycling endosomes.^12^ We next investigated whether VAMP3 was involved in this process. Indeed, there was enhanced colocalization of VAMP3 with ATG16L or GFP-LC3B (Figures S3G–S3J) after CEMM disruption when compared with the control or cholesterol-replenished cells, indicating that CEMMs may have negative effects on recruitment of VAMP3 to the ATG16L1-positive and LC3B-positive vesicles.

**Figure 3:**
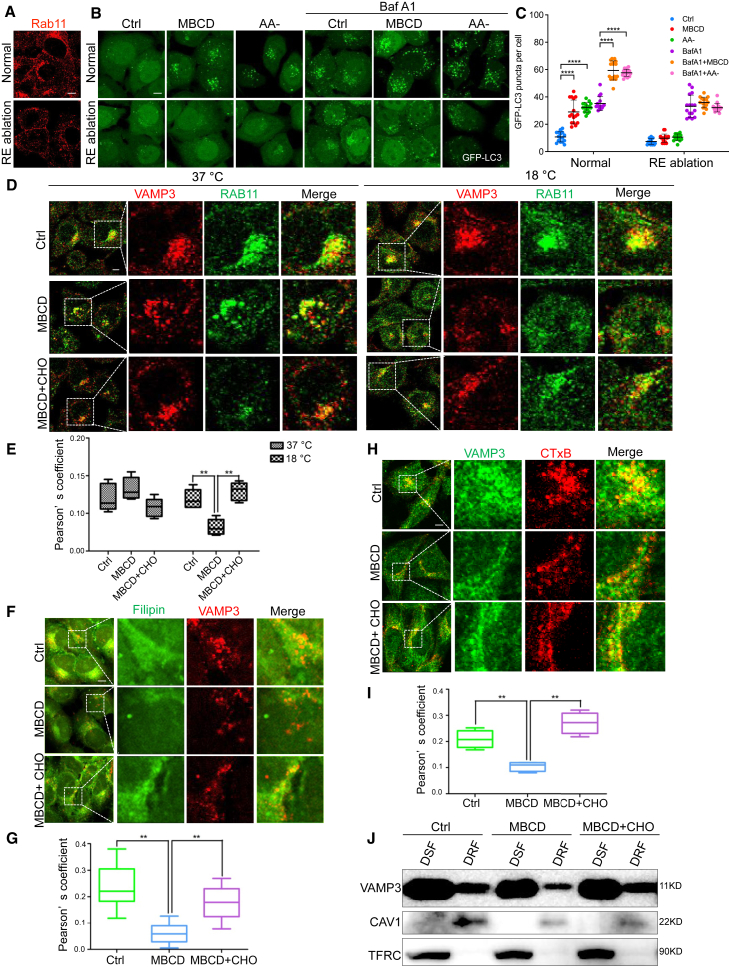
CEMM disruption releases VAMP3 from CEMMs at recycling endosomal membrane (A) After ablation of recycling endosomes, HeLa cells were immunostained with Rab11 (red), and observed under a confocal microscope (×600). Scale bars, 5 μm. (B) After ablation of recycling endosome, HeLa cells with stable expression of GFP-LC3B were pre-treated with MBCD (5 mM, 1 h) and then incubated in the presence or absence of Baf A1 (100 nM). Then cells were observed under a confocal microscope (×600). Scale bars, 5 μm. (C) The number of GFP-LC3 puncta observed in (B) are presented as means ± SD. ∗∗∗∗p < 0.0001. (D) HeLa cells were pre-treated with MBCD (5 mM, 1 h) and then incubated in the presence or absence of CHO (30 μg/mL) at 37°C or 18°C. Cells were immunostained by RAB11 (green) and VAMP3 (red). Scale bars, 5 μm. (E) The Pearson correlation coefficient of RAB11 and VAMP3 from the experiment described in (D) was summarized to represent the colocalization efficiency. ∗∗p < 0.01. (F) HeLa cells were pre-treated with MBCD (5 mM, 1 h) and then incubated in the presence or absence of CHO (30 μg/mL). Then cells were stained with Filipin (excitation, 365 nm; emission, 397 nm; false colored green) and then immunostained by VAMP3 (red). Scale bars, 5 μm. (G) The Pearson correlation coefficient of Filipin with VAMP3 from the experiment described in (F) was summarized to represent the colocalization efficiency. ∗∗p < 0.01. (H) HeLa cells were treated as described in (F). Then cells were stained with CTxB (red) and then immunostained by VAMP3 (green). Scale bars, 5 μm. (I) The Pearson correlation coefficient of CTxB with VAMP3 from the experiment described in (H) was summarized to represent the colocalization efficiency. ∗∗p < 0.01. (J) HeLa cells were treated as described in (F). Then cells were fractioned into a detergent soluble fraction (DSF) and a detergent-resistant fraction (DRF). Both lysates were separated and immunoblotted with the indicated markers.

To further test the involvement of recycling endosomes in CEMM disruption-induced autophagy, we utilized an established recycling endosome ablation approach by using combined treatment with 3,3′-diaminobenzidine (DAB) and H_2_O_2_ to the cells pre-loaded with horseradish peroxidase-transferrin (HRP-TF).^60^^,^^61^ The significant reduction of the RAB11 signaling in the recycling endosome-ablated cells (RE ablation) proved the ablation efficiency of this method (Figure 3A). Importantly, CEMM disruption-induced autophagic flux is almost totally blocked by RE ablation (Figures 3B and 3C), indicating the importance of recycling endosomes in CEMM disruption-induced autophagosome formation.

Next, we aimed to understand whether CEMMs presented in recycling endosomes change the distribution of VAMP3 and thus influence the subsequent formation of autophagosomes. Unexpectedly, we found that MBCD treatment or cholesterol replenishment did not cause any significant changes of the distribution pattern of the VAMP3 and recycling endosome marker RAB11 under normal culturing conditions (Figures 3D and 3E, left panel).^60^ This phenomenon might be caused by the continuing supplements of VAMP3 protein from early endosomes to recycling endosomes.^12^^,^^62^ Therefore, we incubated cells at 18°C to inhibit membrane trafficking between early to recycling endosomes.^63^^,^^64^ As expected, the low temperature caused a decrease in the colocalization between RAB11 and VAMP3 after CEMM disruption (Figures 3D and 3E, right panel), suggesting that CEMMs are involved in the regulation of VAMP3 distribution on recycling endosomes.

Interestingly, VAMP3 is also reported to be partially enriched in the CEMM fraction in macrophages.^65^ Here, we confirmed the enrichment of VAMP3 in CEMM by observing colocalization of VAMP3 with the CEMM markers CTxB and Filipin (Figures 3F–3I). Interestingly, disruption of CEMM by MBCD evidently reduced such colocalization, which was abolished by cholesterol replenishment (Figures 3F–3I). We further confirmed the presence of VAMP3 in CEMM fractions isolated by non-ionic detergents. We found that, although more VAMP3 was found in the DSF (detergent soluble fraction, non-CEMM fraction), treatment with MBCD was able to reduce the VAMP3 level in the DRF (detergent-resistant fraction, CEMM fraction) (Figure 3J). Altogether, these results suggest that disruption of CEMMs releases VAMP3 from recycling endosomes, which is linked to autophagosome biogenesis.

### Inhibition of VAMP3 activity decreases autophagosome biogenesis induced by CEMM disruption

**Figure 4:**
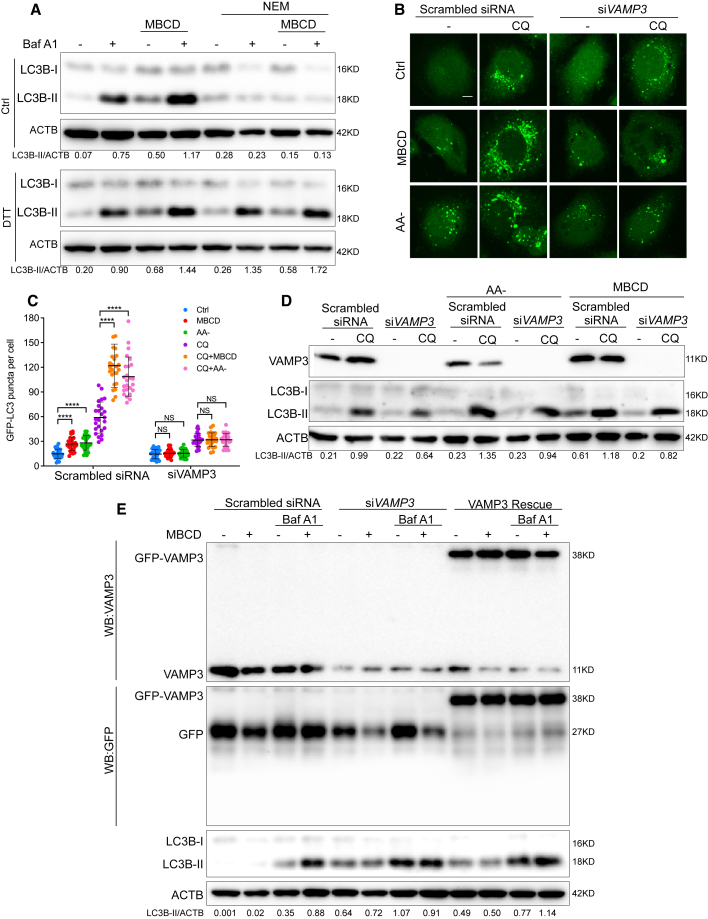
Inhibition of VAMP3 activity decreases autophagosome biogenesis induced by CEMM disruption (A) HeLa cells were pre-treated with 100 μM N-ethylmaleimide (NEM) in the absence or presence of 200 μM dithiothreitol (DTT) on ice for 30 min. Then the cells were incubated with normal medium or medium containing MBCD (5 mM) for 1 h and following treatments as indicated for 2 h, cell lysates were collected and subjected to western blots for the indicated markers. (B) HeLa cells with stable expression of GFP-LC3 were transfected with scrambled siRNA or *VAMP3* siRNAs (si*VAMP3*) for 24 h. Then cells were pre-treated with MBCD (5 mM) for 1 h, and following incubated with or without CQ (50 μM) for 2 h. GFP-LC3 puncta were observed under a confocal microscope (×600). Scale bars, 5 μm. (C) The number of GFP-LC3B puncta observed in (B) are presented as means ± SD. ∗∗∗∗p < 0.0001; NS > 0.05. (D) HeLa cells with the same treatments as described in (B) were harvested and examined by western blots. (E) HeLa cells were transfected with scrambled siRNA or *VAMP3* siRNAs (si*VAMP3*) for 24 h. Then the cells were transfected with EGFP or EGFP-VAMP3 to rescue VAMP3 expression. The cells were pre-treated by MBCD (5 mM) for 1 h, and subsequently incubated with or without Baf A1 (100 nM) for 2 h. The cell lysates were collected and subjected to western blots for the indicated markers.

We next intended to study whether the activity of VAMP3 is required for CEMM disruption-induced autophagosome biogenesis. To do this, we inhibited the function of VAMP3 with N-ethylmaleimide (NEM), a well-established inhibitor for SNARE proteins by blocking disassembly of the SNAREs complex.^66^ The presence of NEM significantly reduced the LC3B-II levels in cells treated with MBCD and Baf A1 (Figure 4A), indicating that NEM is effective in blocking CEMM-induced autophagosome biogenesis/autophagic flux. Meanwhile, when recovering SNAREs protein function by pre-treatment with dithiothreitol (DTT), which is known to quench NEM,^67^ DTT effectively restored LC3B-II levels abolished by NEM in cells treated with MBCD (Figure 4A). Our results thus confirm the function of SNAREs in autophagosome biogenesis induced by CEMM disruption. To specifically validate the importance of VAMP3, we used siRNA targeting *VAMP3*. *VAMP3* knockdown (KD) also markedly reduced GFP-LC3 puncta and LC3B lipidation in cells treated with MBCD in the presence of Baf A1 or CQ (Figures 4B–4E), which could be rescued by overexpression of EGFP-VAMP3 (Figure 4E), further confirming the critical role of VAMP3 in autophagosome biogenesis induced by CEMM disruption.

### Decreased VAMP3-STX6 interaction by CEMM disruption promotes autophagosome biogenesis

**Figure 5:**
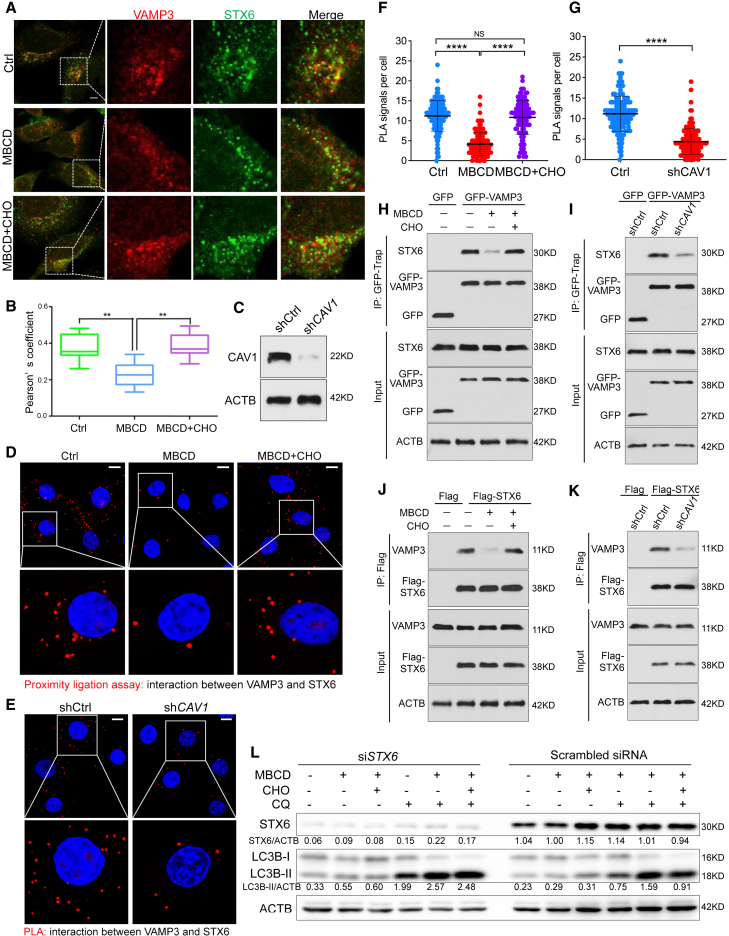
Decreased interaction between VAMP3 and STX6 by CEMM disruption promotes autophagosome biogenesis (A) HeLa cells were pre-treated with MBCD (5 mM, 1 h) and then incubated in the presence or absence of CHO (30 μg/mL). Cells were immunostained by STX6 (green) and VAMP3 (red). Scale bars, 5 μm. (B) The Pearson correlation coefficient of VAMP3 with STX6 from the experiment described in (A) was summarized to represent the colocalization efficiency. ∗∗p < 0.01. (C) HeLa cell with transit transfection of shCtrl or sh*CAV1* were blotted for indicated markers. (D) HeLa cells were treated as described in (A), then were fixed and followed by PLA to detect the interaction between VAMP3 and STX6. The nuclei were counterstained with DAPI. Representative PLA images were selected and are shown. Scale bars, 10 μm. (E) HeLa cells with transit transfection of shCtrl or sh*CAV1* were fixed and followed by PLA to detect the interaction between VAMP3 and STX6. The nuclei were counterstained with DAPI. Scale bars, 10 μm. (F) PLA signals per cell from the experiment described in (D) are summarized. ∗∗∗∗p < 0.0001; NS > 0.05. (G) PLA signals per cell from the experiment described in (E) are summarized. ∗∗∗∗p < 0.0001. (H) HeLa cells with expression of GFP vector or GFP-VAMP3 were treated as described in (A). Then the cell lysates were immunoprecipitated with GFP-trap beads and analyzed for co-immunoprecipitation of STX6. (I) shCtrl and sh*CAV1* MDA-MB-231 cells with expression of GFP vector or GFP-VAMP3 were collected and immunoprecipitated with GFP-trap beads and analyzed for co-immunoprecipitation of STX6. (J) HeLa cells with expression of Flag vector or Flag-STX6 were treated as described in (A). Then the cell lysates were immunoprecipitated with anti-Flag antibody and analyzed for co-immunoprecipitation of VAMP3. (K) shCtrl and sh*CAV1* HeLa cells with expression of Flag vector or Flag-STX6 were collected and immunoprecipitated with anti-Flag antibody and analyzed for co-immunoprecipitation of VAMP3. (L) HeLa cells were transfected with scrambled siRNA or *STX6* siRNAs (si*STX6*) for 24 h. Then cells were pre-treated with MBCD (5 mM) for 1 h, and subsequently incubated with or without CQ (50 μM) for 2 h. Cells were collected and immune-blotted with the indicated markers.

To further explore the molecular mechanism underlying the regulatory role of CEMMs in VAMP3 function, we examined the distribution of STX6, a SNARE protein that is known as a cholesterol-binding protein and a binding partner of VAMP3.^68^^,^^69^ Consistent with previous reports, the interaction between STX6 and VAMP3 was confirmed by their colocalization in cells with normal cholesterol and proficient CAV1 levels (Figures 5A and 5B). Then we performed a positive proximity ligation assay (PLA) signal in HeLa cells after cholesterol manipulation or sh*CAV1* KD (Figures 5C–5G). This interaction was further confirmed by co-immunoprecipitation in the cells with transit expression of GFP-VAMP3 or Flag-STX6 (Figures 5H–5K). Next, we confirmed the distribution of STX6 in CEMM by its colocalization with Filipin staining (Figures S4A and S4B) and its presence in the DRF (Figure S4C). Notably, the distribution of STX6 in CEMMs was reduced by MBCD treatment and recovered by cholesterol replenishment (Figures S4A–S4D).

Second, in STX6 KD cells, MBCD is much less effective in increasing the autophagy flux, meanwhile cholesterol replenishment failed to reduce LC3B puncta formation and lipidation (Figure 5L). These data suggest that the regulatory role of CEMM in autophagy is associated with STX6. However, unlike VAMP3, colocalization between GFP-LC3 and STX6 itself was barely observed in the cells with or without CEMM disruption (Figures S4E and S4F), indicating that the STX6 *per se* might not be directly involved in autophagosome formation. Thus, we believe that the VAMP3 released from its interaction with STX6 might be the key in autophagy induced by CEMM disruption.

### CEMM deficiency-induced autophagy is associated with the acquisition of Doxo resistance in breast cancer cells

**Figure 6:**
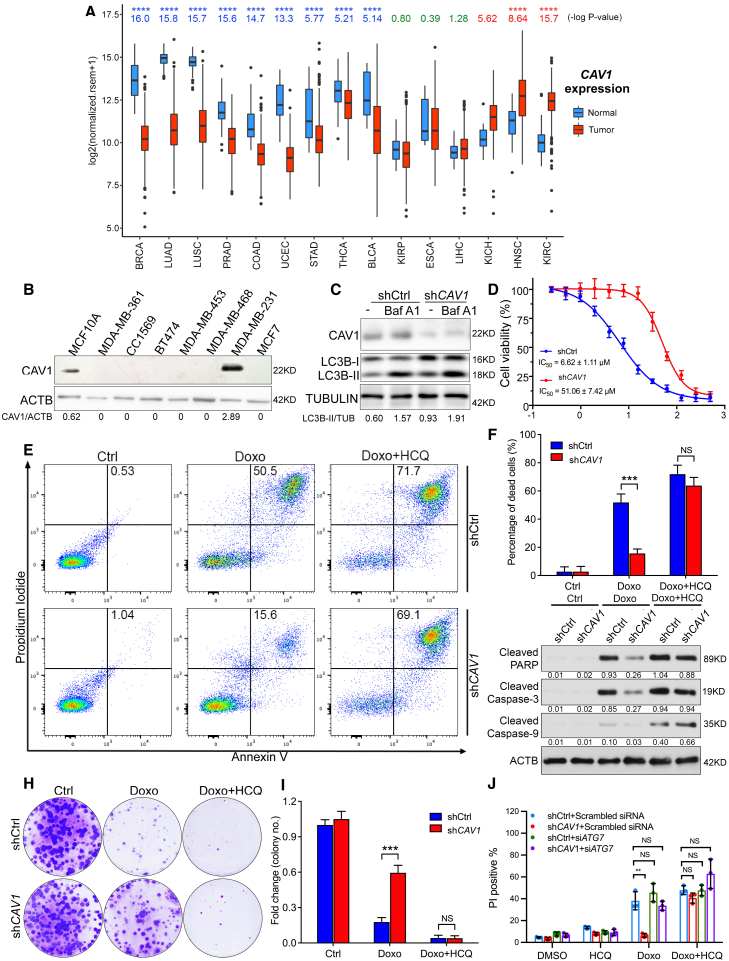
CEMM deficiency-induced autophagy is associated with the acquisition of Doxo resistance in breast cancer cells (A) The expression levels of CAV1 were detected from the TCGA databases in different types of tumor samples and paired normal tissues. Red bars, tumor samples; blue bars, normal samples. ∗∗∗∗p < 0.0001. (B). CAV1 expression in mammary epithelial cell lines MCF10A and seven different breast cancer cell lines. (C) Dox-on shCtrl and sh*CAV1* MDA-MB-231 cells in the presence of Dox in culture medium were treated with or without Baf A1 (100 nM) as indicated for 2 h. Then cells were collected and immunoblotted with the indicated markers. (D) The sulforhodamine cytotoxicity assay to evaluate Doxo in shCtrl and sh*CAV1* MDA-MB-231 cells. shCtrl and sh*CAV1* MDA-MB-231 cells were treated with an increasing concentration of Doxo for determination of growth inhibitory IC_50_ values. The data were normalized against 100% survival at the lowest inhibitor concentration. Graphs for IC_50_ were fitted to the four-parameter logistic equation using Prism8 and shown in the table. Error bars show the percent coefficient of variation. (E) shCtrl and sh*CAV1* MDA-MB-231 cells were treated with Doxo (5 μM), HCQ (100 μM), or their combination for 48 h. After treatments, cells were collected and stained with PI and annexin V and then subjected to flow cytometry. (F) Statistical analysis of experiments described in (E) are presented. ∗∗∗p < 0.001; NS > 0.05. (G) shCtrl and sh*CAV1* MDA-MB-231 cells were treated as described in (E). Then cells were collected and examined with the indicated markers by western blots. (H) Colony formation assay for the shCtrl and sh*CAV1* MDA-MB-231 cells treated as indicated for 48 h. (I) Statistical analysis of experiments described in (H) are presented. ∗∗∗p < 0.001; NS > 0.05. (J) Dox-on shCtrl and sh*CAV1* MDA-MB-231 cells were incubated in the presence Dox in culture medium. After transfection with *ATG7* siRNA (si*ATG7*) or scrambled siRNA for 48 h, cells were treated with Doxo (5 μM), HCQ (100 μM), or their combination for 48 h. Then cells were stained with PI (red) and observed under a fluoresce microscope. The stable shCtrl and sh*CAV1* MDA-MB-231 cells expressed GFP protein when the Dox-on element is activated. Statistical analysis of observed percentage of PI-positive cells is presented. ∗∗p < 0.01; NS > 0.05.

Since we reported the downregulation of CEMMs and enhanced autophagy level were observed in human breast cancer cells and tissues,^32^ here we further analyzed the expression of CEMM marker protein CAV1 in both normal and tumor tissues from TCGA database. We found the CAV1 expression is significantly decreased in 9 of the 15 cancer types, which contain enough data for this kind of analysis (Figure 6A). Breast invasive carcinomas (BRCA) have the most dramatic decrease of CAV1 in cancer tissues (Figure 6A). This is further supported by the finding that CAV1 protein levels are almost undetectable in most breast cancer cell lines (except for MDA-MB-231 cells) (Figure 6B). In addition, we found the CAV1 downregulation is significantly associated with poor prognosis in patients with breast cancer by log rank test in the clinical data from Gene Expression Omnibus (GEO) datasets (GSE1456, 159 patients; GSE3494, 251 patients) (Figures S5A–S5D). Thus, we are curious to know whether autophagy is induced and plays a pro-survival function in those CAV1-deficient breast cancer cells.

To address this question, we established a doxycycline (Dox)-inducible CAV1 KD MDA-MB-231 cell line (the only breast cancer cell line we tested with normal CAV1 expression) and confirmed that the basal autophagy level is indeed promoted by CAV1 deficiency (Figure 6C). Interestingly, we found that the CAV1-deficient cells developed a dramatic resistance to Doxo (a first-line drug for breast cancer) when compared with CAV1-proficient cells (from half-maximal inhibitory concentration [IC_50_] = 6.62 to IC_50_ = 51.06) (Figure 6D). Therefore, we then tested whether the autophagy contributed to the Doxo resistance in CAV1-deficient breast cancer cell by using an autophagy inhibitor, HCQ (the only clinically approved autophagy inhibitor) and knock down of an essential gene for autophagy, ATG7.^70^ Consistent with our hypothesis, by detecting cell death by flow cytometry after propidium iodide (PI) and annexin V labeling (Figures 6E and 6F) and examining apoptosis marker cleaved PARP, caspase-3, and caspase-9 (Figure 6G), we found that *CAV1* KD cells were more resistant to Doxo treatment compared with *CAV1*-proficient cells, which was attenuated by further addition of HCQ. This result is further confirmed by colony formation assay (Figures 6H and 6I). In addition, knock down of *ATG7* impedes the Doxo resistance in *CAV1* KD cells (Figures 6J and S5E). Addition of HCQ did not further enhance cell death in Doxo-treated *ATG7* KD cells, also indicating that the increased cell death caused by Doxo plus HCQ is autophagy dependent. Thus, CAV1 deficiency induces drug resistance against Doxo, and combination with the autophagy inhibitor HCQ overcomes such resistance.

### Autophagy inhibitor HCQ overcomes Doxo resistance in CAV1 KD breast tumors

**Figure 7:**
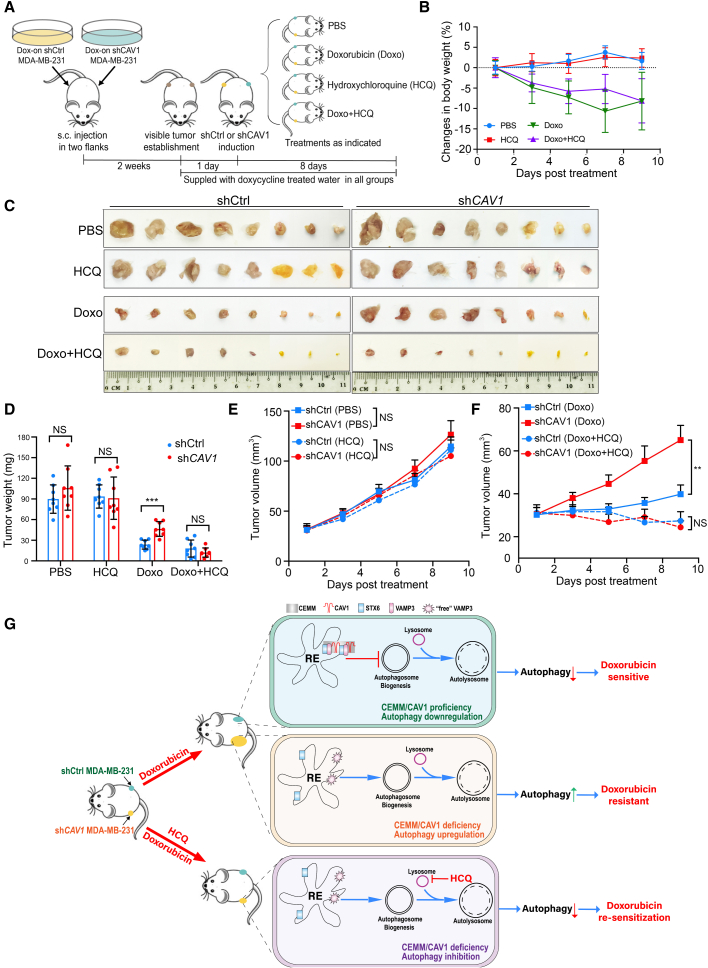
Autophagy inhibitor HCQ overcomes Doxo resistance in *CAV1* KD breast tumors (A) Illustration of the mice xenograft model. Two weeks after subcutaneous implantation of 2 × 10^6^ Dox-on sh*Ctrl* and Dox-on sh*CAV1* MDA-MB-231 cells in both sides of the flanks of female SCID mice were fed with Dox-treated water starting 1 day before administration of the following treatments: (1) PBS, (2) Doxo 6 mg/kg/week, (3) HCQ 50 mg/kg/day, or (4) Doxo plus HCQ via i.p. injection. After 8 days of administration, tumors were isolated, and tumor volumes were examined and estimated every 2 days. (B) Changes in body weight of different groups were presented as mean ± SE. (C) The xenograft tumor collected and presented in different treatment groups. (D) Tumor mass was demonstrated by mean ± SE. ∗∗∗p < 0.0001; NS > 0.05. (E and F) Changes of mean tumor volume of indicated groups are presented. ∗∗p < 0.01; NS > 0.05. (G) CEMM/CAV1 deficiency promotes autophagosome biogenesis and increases Doxo resistance in breast tumors. (1) In CEMM/CAV1-proficient breast tumors, CEMMs in recycling endosomes support VAMP3-STX6 interaction and function as barriers to limit the positive role of VAMP3 in autophagosome biogenesis. Thus, autophagy is maintained at a relatively low level. Tumors are sensitive to Doxo treatment. (2) In CEMM/CAV1-deficient breast tumors, decreasing CEMMs by CAV1 deficiency promotes Doxo resistance via upregulation of autophagy. (3) In CEMM/CAV1-deficient breast tumors with autophagy inhibition by HCQ, blockage of autophagy leads to Doxo re-sensitization and thus significantly reduced tumor loads.

In the next step, we established a xenograft mice model to confirm that the effect of Doxo and HCQ combination in CAV1 downregulated breast cancer cells. Female severe combined immunodeficiency (SCID) mice, 8 weeks old, were inoculated subcutaneously in both sides of their flanks with Dox-on shCtrl wild type (WT) and Dox-on sh*CAV1* MDA-MB-231 cell KD to establish tumors. Two weeks after inoculation, mice bearing visible tumors were fed with Dox-treated water to induce CAV1 KD in tumors originating from sh*CAV1* cells, and tumors originating from shCtrl maintained proficient CAV1 expression. Then mice were then randomly assigned to four groups with the following treatments: vehicle control, PBS; HCQ; Doxo; and Doxo plus HCQ (Figure 7A). The changes in body weight in the different groups were within 10% (Figure 7B). We found that Doxo treatment was significantly less effective in the *CAV1* KD tumor compared with WT tumors, indicating that CAV1 deficiency promotes resistance to Doxo treatment (Figures 7C–7F). Importantly, combination of the autophagy inhibitor HCQ with Doxo dramatically diminishes the superiority of the *CAV1* KD tumors (Figures 7C–7F), suggesting a possible stratagem to overcome Doxo resistance in CAV1-downregulated breast cancer patients by targeting autophagy.

## Discussion

In this study, we provide sufficient evidence to show that CEMM deficiency promotes autophagosome biogenesis and increases Doxo resistance in breast tumors. Mechanically, we reveal a novel function of CEMMs in regulating autophagy at the early stage: CEMMs negatively regulate autophagosome biogenesis via modulation of interaction between STX6 and VAMP3. Upon CEMM disruption, recycling endosomes-containing VAMP3 are released from STX6 and re-distribute to autophagic vesicles to promote the coalescence of autophagosome membrane vesicles for autophagosome biogenesis. Following this scenario, breast tumors with CEMM/CAV1 deficiency utilize autophagy as a pro-survival mechanism responding to Doxo treatment (Figure 7G). Thus, inhibition of autophagy could be a useful strategy for these patients with low *CAV1* expression and resistance to Doxo treatment.

Our findings are generally consistent with early reports that CEMM or its major component cholesterol are considered as a negative regulator of autophagy.^32^, ^33^, ^34^, ^35^, ^36^, ^37^, ^38^, ^39^, ^40^^,^^71^ We demonstrate the importance of recycling endosomes on autophagosome biogenesis induced by CEMM disruption. Recycling endosomes are known to “feed” autophagosome by supplying membrane for autophagosome formation.^12^^,^^60^^,^^72^^,^^73^ In this study, we identified the existence of CEMM on recycling endosomes (Figures S3A and S3B), which is consistent with previous report.^59^ Second, we found this recycling endosomes-containing CEMMs provide a platform for the interaction between STX6 and VAMP3, which functions as barriers for VAMP3 activation and subsequent autophagosome formation (Figures 3, 4, and 5). Interestingly, our previous study showed the association between lysosome-containing CEMM and V-ATPase (v0 subunits) contributes to autophagy suppression.^32^ These findings thus suggest that CEMM presented at different subcellular organelles possess similar negative effect on autophagy. However, it is known CEMM present at plasma membrane generally act as a positive mechanism in signal transduction or membrane trafficking by providing a platform for various important receptors or adaptor proteins.^23^^,^^74^, ^75^, ^76^ Thus, the negative regulatory role of CEMM in autophagy at both the early and late stage reveals a novel spectrum of CEMM functions in cell biology.

Here, we found the accumulation of VAMP3 and STX6 on CEMM in the untreated control cells (Figures 3F–3J and S4A–S4C), which is consistent with previous reports showing enrichment of SNAREs proteins at CEMMs.^71^^,^^77^, ^78^, ^79^ With the exception of localization of SNAREs, the function of SNAREs is also known to associate with CEMMs, although how the functions of the SNAREs are regulated by CEMMs remains largely elusive and often controversial.^71^^,^^77^, ^78^, ^79^, ^80^, ^81^ On one hand, the enriched SNAREs in CEMMs have been found to be required for efficient fusion events during endocytosis, indicating a positive role of CEMM in promoting SNAREs activity.^82^, ^83^, ^84^ On the other hand, CEMM may suppress the autophagosome-lysosome fusion process mediated by SNAREs.^71^ Here, we show that CEMMs play a positive role in maintaining the VAMP3-STX6 interaction, via which CEMMs are able to retain VAMP3 in recycling endosomes and thus prevent the pro-autophagic function of VAMP3. This scenario is consistent with a previous study that shows that the accumulation of cholesterol in recycling endosomes results in STX6 accumulation and its interaction with VAMP3, which subsequently blocks the recycling of αVβ3 and α5β1 integrins and cell migration.^68^ Therefore, we believe that the CEMMs play a dual role in SNARE regulation: CEMMs can be considered as platforms or barriers for different SNARE functions in different organelles. Furthermore, the major component of CEMMs, cholesterol, has also been reported to play an important role in regulation of localization and function of SNAREs. Eleven of 38 SNAREs found in human are known to interact with cholesterol through their cholesterol-binding motifs.^69^ Our finding is consistent with a previous study in which the modulation of cholesterol levels at the *trans-*Golgi network and recycling endosome membrane regulates STX6 localization and its interaction with VAMP4 or VAMP3.^68^

Notably, Nozawa et al. have identified the positive regulatory role of the STX6-VTI1B-VAMP3 complex on xenophagy recently.^85^ They reported the localization of VAMP3 on recycling endosomes and the interaction between STX6 and VAMP3, which is generally consistent with our findings (Figures 3D, 3E, and 5A–5J). Different from their study, our data show that the disassociation between STX6 and VAMP3 contributes to MBCD-induced autophagosome biogenesis (Figures 4, 5, and S4). Different membrane trafficking situations may explain such discrepancies. In our system, MBCD disrupts CEMMs in the intracellular membrane system, while, during xenophagy, bacterial pathogens carry secreted toxins that interact with cholesterol and may supply more cholesterol to the intracellular system.^86^^,^^87^ Therefore, the cholesterol-sensitive STX6-VAMP3 complex might function distinctively in response to different intracellular cholesterol levels. Further study is required to confirm this hypothesis.

Consistent with previously literature, by analyzing clinical databases, and confirmation in different breast cancer cell lines, we report downregulated CEMM protein CAV1 in breast cancer and its association with poor clinical prognosis (Figures 6A, 6B, and S5A–S5D).^88^, ^89^, ^90^ We firstly linked CAV1 downregulation to Doxo resistance in breast cancer treatment.^91^ Doxo is one of the most commonly used chemotherapeutics in breast cancer treatment. However, the development of drug resistance to Doxo impedes its effect on chemotherapy.^91^ We found that *CAV1* KD in a *CAV1*-proficient breast cancer cell line MDA-MB-231 significantly reduces cell death caused by Doxo in an autophagy-dependent manner (Figures 6D–6J). By establishing a mouse xenograft model for inducible *CAV1* KD tumors, we confirmed that the autophagy inhibitor HCQ dramatically overcomes Doxo resistance in *CAV1* KD tumors (Figures 7A–7F), suggesting that autophagy might be a potential target for patients with low CAV1 expression and Doxo resistance. Thus, CAV1 expression levels could be used as a biomarker for autophagy inhibitory strategy in cancer treatment.

Taken together, we provide clear evidence revealing a novel mechanism underlying the negative regulatory function of CEMMs on autophagy: CEMMs in recycling endosomes support VAMP3-STX6 interaction and function as barriers to limit the positive role of VAMP3 in autophagosome biogenesis. Downregulation of CEMM by CAV1 deficiency is associated with poor clinical outcome in breast cancer patients and might be involved in the Doxo resistance. Blockage of autophagy may be a possible solution for Doxo-resistant breast cancer patients with reduced CAV1 expression.

## Materials and methods

### Reagents and antibodies

The chemicals used in this study were: MBCD (Sigma, C4555), cholesterol-water soluble (Sigma, C4951), Baf A1 (Santa Cruz, CAS 88899-55-2), Wort (Santa Cruz, CAS 19545-26-7), cholera toxin subunit B conjugated with Alexa Fluor 594 (CTxB, Invitrogen, C34777), DAB (Dako, K401011), NEM (Sigma, E3876), DTT (Sigma, D9779), human transferrin peroxidase (Rockland antibodies and assays, 009-0334). The antibodies used were: anti-MAP1LC3B/LC3B (Sigma, L7543), anti-ACTB/β-actin (Sigma, A5441), anti-TFRC/TFRC (Invitrogen, 136,800), anti-VAMP3 (Santa Cruz, sc-514843), anti-CAV1/caveolin-1 (BD Pharmingen, 610,060), anti-ATG9A (Abcam, ab108338), anti-RAB11 (Cell Signaling Technology, 5589), mouse anti-STX6 (Invitrogen, 701,823), WIPI2 (Abcam, ab105459), and ATG16L1 (Cell Signaling Technology, 8089).

### Cell lines and cell culture

All the cell lines were grown in Dulbecco’s modified Eagle's medium (DMEM; Sigma, D7777) with 10% fetal bovine serum (Hyclone, SH30071.03), and 1% penicillin-streptomycin (Pan-Biotech, P06-07100) (defined as normal medium in this study) in a 5% CO_2_ incubator at 37°C. The *Atg5* Tet-off inducible MEFs (m5-7) with stable GFP-LC3B expression and HeLa cells with stable expression of GFP-LC3B were kind gifts from Dr. N. Mizushima (University of Tokyo).^92^

### Transient siRNA transfection

*VAMP3*, *STX6*, and *ATG7* siRNA (ON-TARGET plus SMARTpool) were transfected by using Lipofactamine 3000 transfection reagent (Invitrogen, L3000075). Then cells were treated and examined 24–48 h later.

### Plasmids and transient transfection

Strawberry-Atg16L1 was a gift from Dr. T. Yoshimori (Osaka University).^93^ GFP-VAMP3 was a gift from Thierry Galli (Addgene plasmid no. 42,310).^94^ FLAG-STX6 (p3XFLAG-CMV 7.1_syn6) was a gift from David Hackstadt (Addgene plasmid no. 50,012).^95^ mCherry-D4H was a gift from Gregory D. Fairn.^52^ Lipofectamine 3000 transfection reagent was used for transient transfection according to the manufacturer's protocol.

### Dox-on shCtrl (WT) and Dox-on sh*CAV1* MDA-MB-231 cells (KD) establishment

SMARTVector Inducible Human *CAV1* shRNA lentiviral particles (tGFP-CMV-sh*CAV1*, targets sequences consisting of V3SH7669–228785932, V3SH7669–230109760, and V3SH7669-225403960) were transduced in cultured MDA-MB-231 cells according to the manufacturer’s protocol (Horizon, SMARTVector). Control shRNA (h) lentiviral particles was also used as a negative control. Continual selection was followed with 5 μg/mL puromycin to establish stable cell lines. The extent of CAV1 depletion was evaluated by western blot.

### Immunostaining

Cells for immunostaining were grown on coverglass chamber slides and treated as indicated. Then cells were fixed in 4% formaldehyde and permeabilized by 0.5% Triton X-100 (Sigma, X100). Samples were blocked by 1% BSA (Sigma, A7906) in PBS and followed the incubation with the indicated primary and secondary antibodies. Images were captured with a confocal microscope (Olympus Fluoview FV1000).

### Western blotting

After the designated treatments, cells were lysed with Laemmli SDS buffer (62.5 mM Tris [pH 6.8], 25% glycerol, 2% SDS [Vivantis, PB0640], phosphatase inhibitor [Thermo Scientific, 78,428], and proteinase inhibitor cocktail [Roche, 11697498001]). Each sample was loaded on SDS-PAGE gel with equal quantity, and then transferred onto a PVDF membrane (Bio-Rad, 162-0177). Membranes were blocked, followed by incubations with the indicated primary and secondary antibodies. Then the membrane was visualized using an enhanced chemiluminescence method (Thermo Scientific, 34076) using the ImageQuant LAS 500 (GE).

### Cholesterol manipulation

For cholesterol depletion, 1 h pre-treatment of 5 mM MBCD was utilized. Then, after washing using PBS, normal DMEM medium with the indicated treatment were added. For cholesterol replenishment, after depletion of cholesterol by 1 h MBCD (5 mM) pre-treatment, the cells were washed by PBS twice and then changed to DMEM medium containing 30 μg/mL cholesterol-water soluble (CHO) with or without the indicated treatment for 2 h. Then the cells were collected to perform the following assays.

### Filipin staining

Filipin III (Sigma, F4767) was used to label CEMM as reported previously.^96^ The cells seeded on coverglass slide changers were treated and fixed with 4% paraformaldehyde for 30 min and quenched in 50 mM NH_4_Cl_3_ for 10 min. Then the solution, which contained 0.2% BSA (Sigma, A7906), 0.2% fish skin gelatin (Sigma, G7041), and 50 μM of Filipin III was used to block, permeabilize, and stain the cells. After 20 min incubation at room temperature, PBS was used to wash the cells (3 times for 5 min). For combination with immunostaining, the Filipin-stained cells were followed by incubation of the indicated primary and secondary antibodies. Confocal microscopy was used to detect the signals.

### CTxB staining

The conjugated with Alexa Fluor 594 (CTxB) staining was done as described previously.^51^ In brief, cells were cultured on coverglass chamber slide. Cells were loaded with 1 μg/mL CTxB for 15 min on 4°C. Then cells were washed and followed by the indicated treatments at 37°C. The cells were examined directly or with subsequent immunostaining. Images were captured with a confocal microscope (Olympus Fluoview FV1000).

### Electron microscopy

HeLa cells with the designated treatments were fixed for 1 h at room temperature with freshly prepared fixative mixture (2% paraformaldehyde + 3% glutaraldehyde) in 0.1 M cacodylate buffer (pH 7.4). After rinsing the cells three times with the same buffer, they were post-fixed with 2% OsO4 (pH 7.4), and block staining was performed as follows: wash twice with 0.1 M PB, dehydrate through an ascending ethanol series (25%, 50%, 75%, 95%, and 100%), and then 2 times in 100% acetone for 10 min. Finally, samples were embedded in fresh resin and polymerized at 60°C for 24 h. Uranyl acetate and lead citrate were used to stain ultrathin sections. After rinsing in distilled water, samples were observed using an electron microscope (JEOL, JEM-1010).

### Cholesterol detection assay

An Amplex Red Cholesterol Assay Kit (Invitrogen, A12216) was used to detect cholesterol concentration in cell lysates according to the manufacturer's protocol.

### Cell fractionation

Isolation of CEMMs by detergent-based or non-detergent-based methods was performed as described previously.^97^, ^98^, ^99^ After the indicated treatments, cells were collected and homogenized with TNE buffer (25 mM Tris-HCl [pH 7.4], 150 mM NaCl, 3 mM EDTA, and a protease and phosphatase inhibitor cocktail) supplied with 1% Triton X-100 (Sigma, X100) by passage through a 27-gauge needle 20 times on ice. Then the cell lysates were centrifuged at 2,000 rpm for 10 min at 4°C to remove nuclear fraction. Afterward, the cell lysates were spun down at 16,000 × *g* at 4°C for 30 min. The supernatants were collected, and the insoluble pellets were resuspended and lysed in Laemmli SDS buffer.

### Co-immunoprecipitation

HeLa cells were transfected with the indicated plasmid, and then collected and homogenized with IP lysis buffer (10 mM Tris-HCl [pH 7.4], 100 mM NaCl, 2.5 mM MgCl_2_, 0.5% Triton X-100, and a protease and phosphatase inhibitor cocktail) through sonication (4 Watt, 5–6 s, 3 cycles). Then the lysates were spun at 10,000 rpm for 2 min at 4°C. The supernatants were pre-cleared with 30 μL protein A/G Agarose beads (Thermo, A/G Agarose beads) for 1 h at 4°C. The pre-cleared supernatants were then incubated with 10 μL GFP-trap or Flag beads (Chromotek, ABIN1082213) overnight at 4°C. The protein-bead complexes were centrifuged at 2,000 rpm for 5 min, and washed with lysis buffer 5 times. Subsequently, they were boiled with Laemmli SDS buffer for 5 min and subjected to western blots.

### PLA

Cells were cultured in coverglass slide chambers and treated as indicated. After fixation with 4% paraformaldehyde and permeabilization with 0.1% saponin (Sigma, 47,036), the cells were subjected to PLA using a Duolink Detection Kit (Olink Bioscience [PLA Probe Anti-Goat MINUS, 92006; PLA Probe Anti-Mouse PLUS, 92001; Detection Reagent Red, 92008]) according to the manufacturer’s instructions.

### BSA-Alexa 488 uptake

The BSA-Alexa 488 uptake assay was performed based on the method described previously.^56^ Cells in 90% confluent were incubated with serum-free DMEM medium for 4 h, and then incubated with the indicated treatments. After this, cells were incubated with 50 μg/mL of BSA- Alexa 488 (Invitrogen, A13100) at 37°C for 0.5 h. The cells were fixed with 4% paraformaldehyde and observed under a confocal microscope.

### Ablation of recycling endosomes

The ablation of recycling endosomes was done as described previously.^60^^,^^61^ Recycling endosomes were pre-loaded with human transferrin peroxidase for 20 min at 37°C. Then the cells were washed and incubated for 1 h with 0.1 mg/mL DAB as a control or 0.1 mg/mL DAB and 0.003% H_2_O_2_ to ablate recycling endosomes labeled with human transferrin peroxidase on 4°C. Afterward, 1% BSA was used to quench the pre-loaded reagents at 4°C. Cells were recovered by incubation at 37°C for 30 min. Finally, cells were treated as indicated and fixed for immunostaining of TFR. Images were captured with a confocal microscope (Olympus Fluoview FV1000).

### Sulforhodamine B cytotoxicity assay

Cells were cultured in 96-well plates, treated as described, and then fixed with trichloroacetic acid. Colorimetric or fluorescence analysis was performed in a FlexStation 3 (Molecular Devices, SoftMax Pro 7.0). Normalized graphs were generated with Prism 8 software, non-linear four-parameter data fitting was performed for calculation of IC_50_ values.

### Colony formation assay

Cells were incubated in 6-well plates and treated as designated, then they were fixed with paraformaldehyde for 20 min, stained by crystal violet for 30 min, and washed with PBS three times.

### MDA-MB-231 tumor xenograft

Under the approval of the Institutional Animal Care and Use Committee of National University of Singapore (R15-0459), we conducted experiments on female SCID mice (6–8 weeks old). Totals of 2 × 10^6^ Dox-on shCtrl (WT) and Dox-on sh*CAV1* MDA-MB-231 cells (KD) were subcutaneously injected into both sides of the flanks, respectively. Two weeks after inoculation, mice bearing visible tumors were fed with 5% sucrose water containing 2 mg/mL Dox/per day and randomly distributed into the following four groups (6 mice/group): (1) PBS, (2) Doxo 6 mg/kg/week, (3) HCQ 50 mg/kg/day, or (4) Doxo plus HCQ via intraperitoneal injection. The body weights and tumor sizes were measured daily. After 8-day treatments, all mice were sacrificed by CO_2_ inhalation. The tumors were isolated and measured.

### CAV1 gene expression analysis

The expression of CAV1 RNA expression in different tumor and normal tissues were analyzed via the Gene Expression Profiling Interactive Analysis (GEPIA) web server based on the GTEx and TCGA projects.^100^

### CAV1 prognosis analysis in breast cancer patients

Publicly available prognosis data were retrieved from the NCBI GEO data repository. We collected breast cancer data from two datasets (GSE1456 and GSE3494). Kaplan-Meier curves were used to compare the overall survival and recurrence-free survival between patients with high and low CAV1 expression. The curves were analyzed using the Kaplan-Meier plotter web server (http://kmplot.com/analysis/index.php?p=background).^101^

### Image analysis

Quantification of puncta was performed using the Analyze particle function of ImageJ software (NIH). The measurements were made on randomly selected fields of view. Colocalization analysis between two channels was performed using the JACoP plugin in ImageJ (NIH).

## Supplemental information

Document S1. Figures S1–S5

Document S2. Article plus supplemental information
