# Supplementary material for: Cholesterol-enriched membrane micro-domaindeficiency induces doxorubicin resistancevia promoting autophagy in breast cancer
Source: Mol Ther Oncolytics. 2021 Oct 20;23:311–29. doi: 10.1016/j.omto.2021.10.005 (PMC8573103; doi:10.1016/j.omto.2021.10.005)
Supplement: Document S1. Figures S1–S5 [file mmc1.pdf]

OMTO, Volume *23*

Supplemental information

Cholesterol-enriched membrane micro-domain deficiency induces doxorubicin resistance via promoting autophagy in breast cancer

Yin Shi, Zu Ye, Guang Lu, Naidi Yang, Jianbin Zhang, Liming Wang, Jianzhou Cui, Miguel A. del Pozo, Yihua Wu, Dajing Xia, and Han-Ming Shen

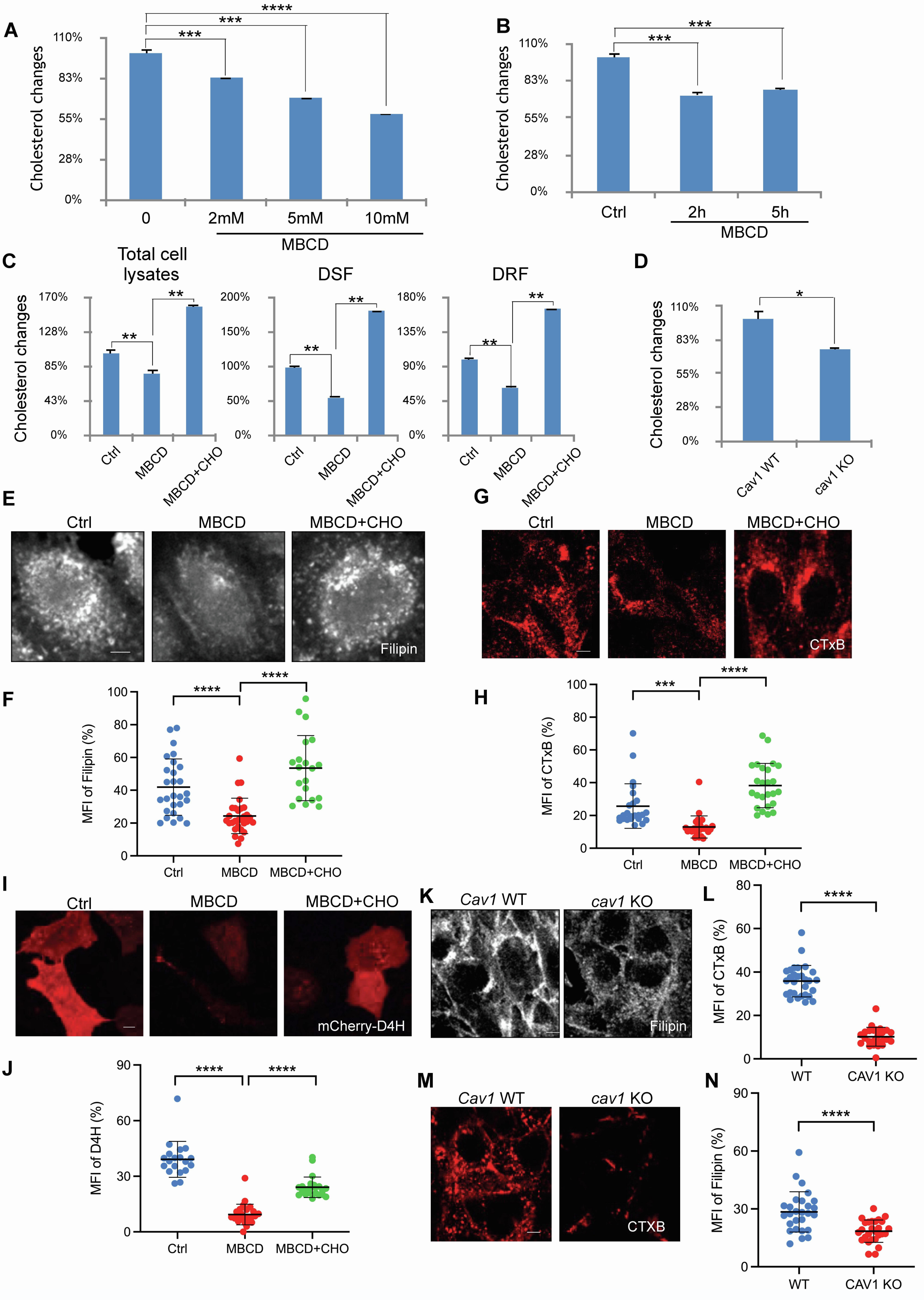

Fig. S1 Disruptive effects of MBCD and *CAV1* knockout on CEMM level. (**A**) HeLa cells were pre-treated with MBCD in indicated concentration for 1 h, then the cholesterol concentration of total cell lysates and different fractions were measured by Amplex® Red Cholesterol Assay Kit. Statistical significance evaluated with a two-tailed Student's t-test. (**B**) HeLa cells were pre-treated with or without MBCD (5 mM) for 1 h, then incubated for indicated time. The cholesterol concentration of total cell lysates and different fractions were measured by Amplex® Red Cholesterol Assay Kit. (**C**) The HeLa cells were treated with MBCD (5 mM) for 1 h, then incubated in the presence or absence of CHO (30 µg/ml). Then cells were fractioned into DSF and DRF. The

cholesterol concentration of total cell lysates and different fractions were measured by Amplex® Red Cholesterol Assay Kit. (**D**) *Cav1* WT and *cav1* KO MEFs lysates were measured by Amplex® Red Cholesterol Assay Kit. (**E**) Cells were treated as described in panel (C), then stained with Filipin (false-colored white) and observed under confocal microscope. Bars, 5 μm. (**F**) The mean fluoresce intensity of Filipin were analyzed by ImageJ. (**G**) HeLa cells were treated as described in panel (C) were stained with CTxB. (**H**) The mean fluoresce intensity of CTxB were analyzed by ImageJ. (**I**) The HeLa cells were transfected with mCherry-D4H, then treated as described in panel (C). (**J**) The mean fluoresce intensity of mCherry-D4H were analyzed by ImageJ. (**K**) *Cav1* WT and *cav1* KO MEFs were stained with Filipin (false-colored white) and observed under confocal microscope. (**L**) The mean fluoresce intensity of Filipin were analyzed by ImageJ. (**M**) *Cav1* WT and *cav1* KO MEFs were stained with CTxB (Red) and observed under confocal microscope. (**N**) The mean fluoresce intensity of CTxB were analyzed by ImageJ.

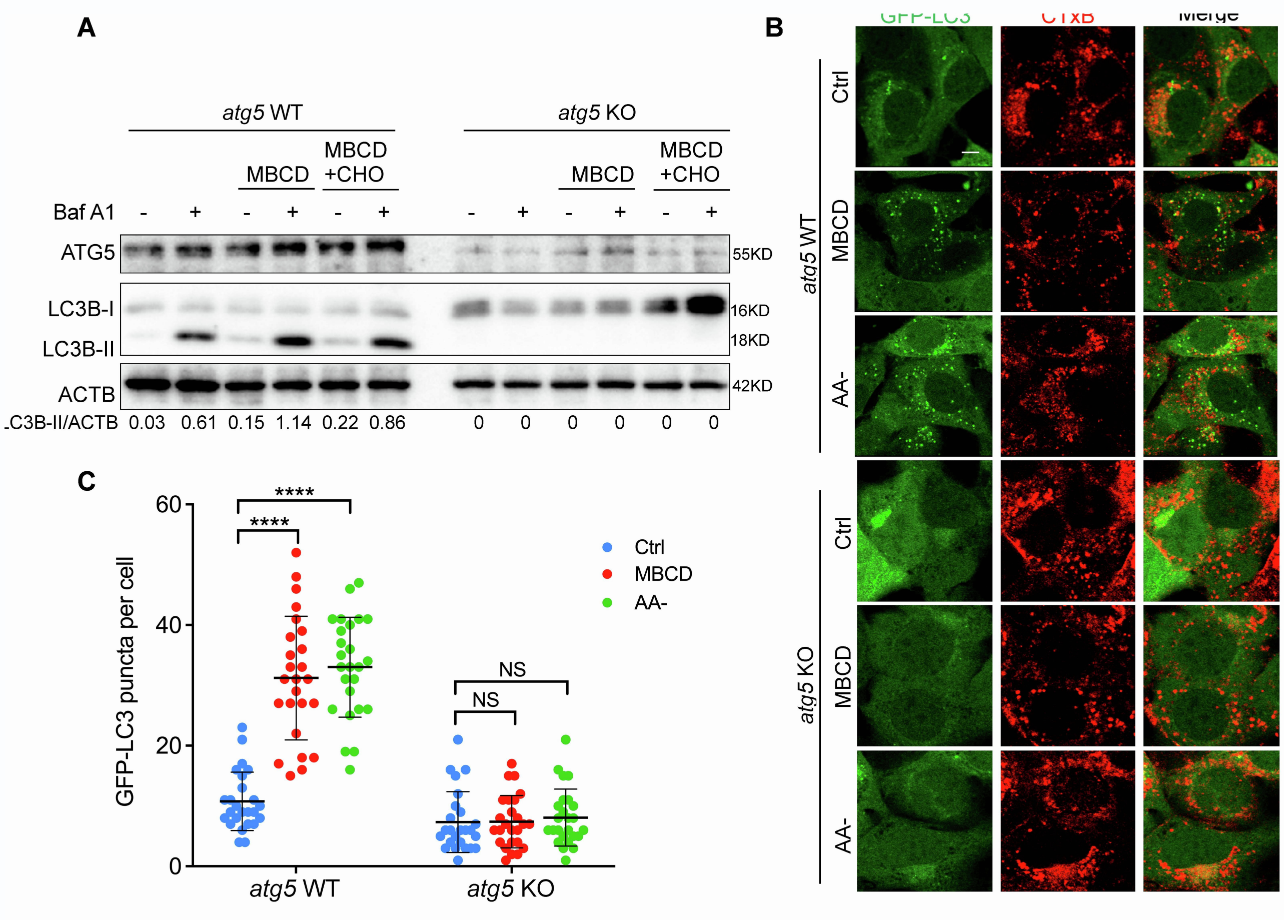

Fig. S2 CEMM disruption-induced autophagy is Atg5 dependent. (**A**) *atg5* WT and KO MEFs with stable expression of GFP-LC3 were pre-treated with or without MBCD (5 mM) for 1 h, then incubated in the presence or absence of cholesterol (CHO, 30 μg/ml) or Baf A1 (100 nM) as indicated for 2 h. Cell lysates were collected and subjected to western blots for the indicated markers. (**B**) *atg5* WT and KO MEFs with stable expression of GFP-LC3 were pre-treated with or without MBCD (5 mM) for 1 h, then incubated in normal or AA- as indicated for 2 h. Cells were stained with CTxB and observed under confocal microscope. (**C**) GFP-LC3 puncta per cell from the experiments described in panel (B) were presented.

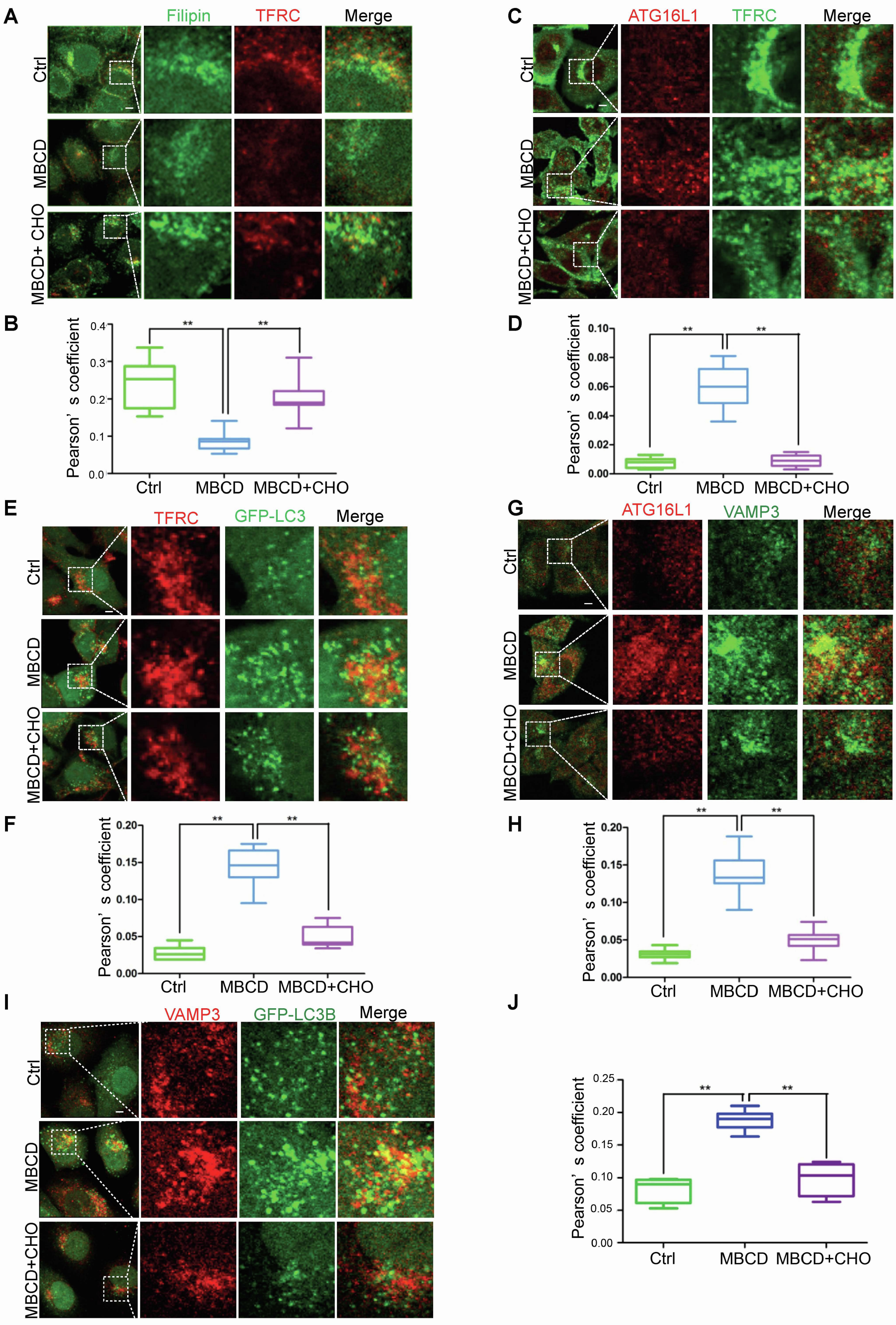

Fig. S3 Recycling endosomes contribute to autophagosome biogenesis induced by CEMM disruption. (**A**) HeLa cells were pre-treated with MBCD (5 mM, 1 h), then incubated in the presence or absence of cholesterol (CHO, 30μg/ml). Then cells were stained with Filipin (false-colored Green) and immunostained by TFRC (Red). (**B**) The Pearson correlation coefficient of Filipin with TFRC from the experiment described in panel (A) was summarized to represent the colocalization efficiency. (**C**) HeLa cells were treated as described in panel (A). Cells were immunostained by TFRC (Green) and ATG16L1 (Red). (**D**) The Pearson's coefficient of ATG16L1 and TFRC in panel (C) are presented as means ± SD and analyzed by statistical analysis. (**E**) HeLa

cells with stable expression of GFP-LC3B (Green) were treated as described in panel (A). Then cells were immunostained by TFRC (Red). (**F**) The Pearson's coefficient of GFP-LC3 and TFRC in panel (E) are presented as means ± SE and analyzed by statistical analysis. (**G**) HeLa cells were treated as described in panel (A). Then cells were immunostained by ATG16L1 (Red) and VAMP3 (Green), and observed under confocal microscope (× 600). (**H**) The Pearson's coefficient of ATG16L1 and VAMP3 in panel (G) are presented. (**I**) HeLa cells with stable expression of GFP-LC3B (Green) were treated as described in panel (A), then immunostained by VAMP3 (Red). (**J**) The Pearson correlation coefficient of VAMP3 with GFP-LC3 from the experiment described in panel (I) was are presented.

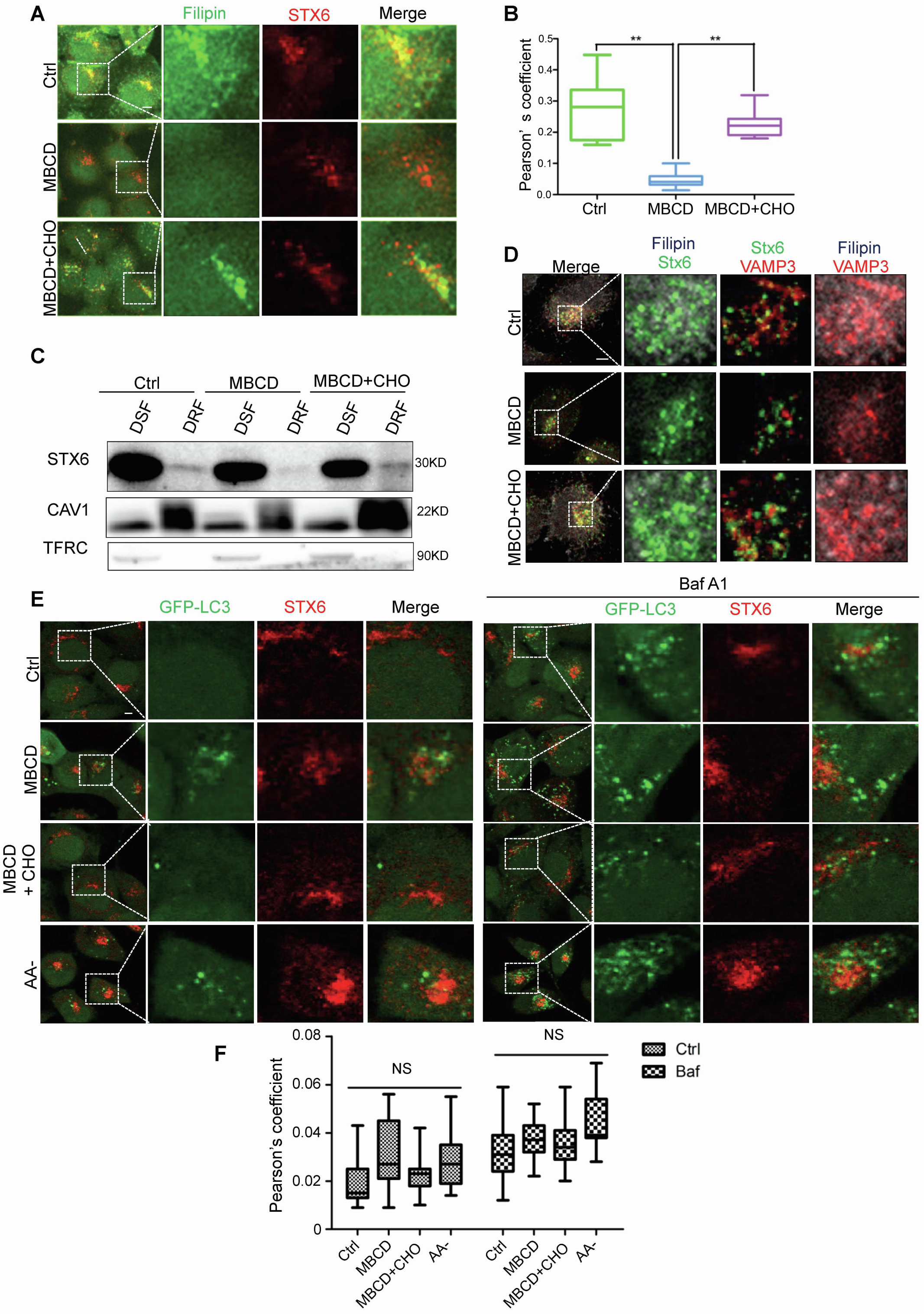

Fig. S4 Decreased interaction between VAMP3 and STX6 by CEMM disruption contributes to autophagosome biogenesis. (**A**) HeLa cells were pre-treated with MBCD (5 mM, 1 h) and then incubated in the presence or absence of CHO (30 μg/ml). Cells were stained with Filipin (false-colored green) and then immunostained by STX6 (Red). (**B**) The Pearson correlation coefficient of Filipin with STX6 from the experiment described in panel (A) was summarized to represent the colocalization efficiency. (**C**) HeLa cells were treated as described in panel (A). Then cells were fractioned into DSF and DRF. Both lysates were separated and immunoblotted with indicated markers. (**D**) HeLa cells treated as described in panel (A) were stained with Filipin (false-colored white) and then immunostained with STX6 (Green) and VAMP3 (Red). Bars, 5 μm. (**E**) HeLa cells with stable expression of GFP-LC3B were treated as indicated. Cells were immunostained by STX6 (Red). (**F**) The Pearson correlation coefficient of STX6 with GFP-LC3B from the experiment described in panel (E) was summarized to represent the colocalization efficiency. Statistical significance evaluated with a two-tailed Student's t-test.

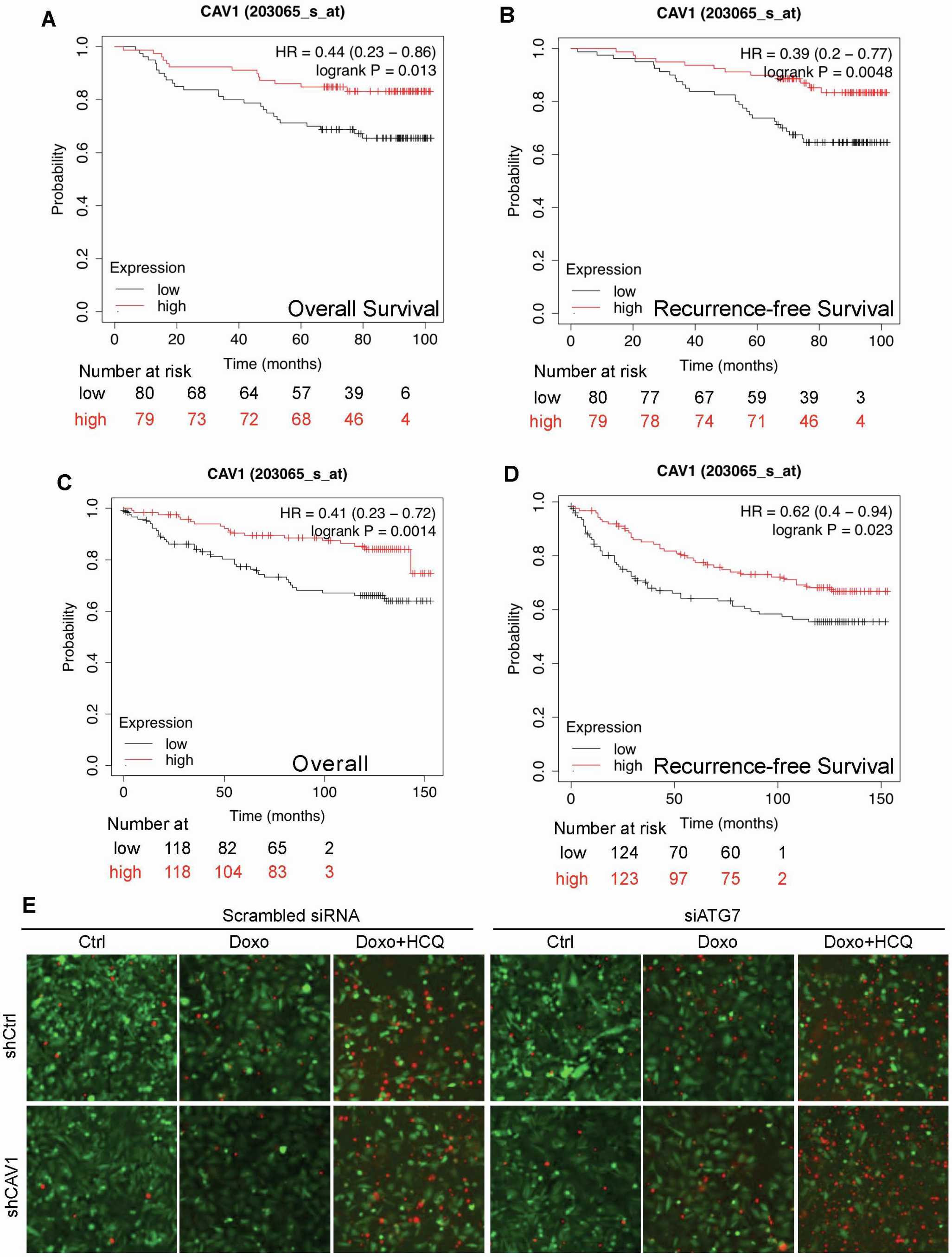

Fig. S5 CEMM deficiency is associated with poor prognosis in patients with breast cancer. (**A**) Kaplan–Meier (KM) curve showing overall survival (OS) in the CAV1 low and CAV1 high groups classified based on the median CAV1 expression level in dataset GSE1456. (**B**) KM curve showing recurrence-free survival (RFS) in the CAV1 low and CAV1 high groups classified based on the median CAV1 expression level in dataset GSE1456. (**C**) KM curve showing OS in the CAV1 low and CAV1 high groups classified based on the median CAV1 expression level in dataset GSE3494. (**D**) KM curve showing RFS in the CAV1 low and CAV1 high groups classified based on the median CAV1 expression level in dataset GSE3494. (**E**) Representative images from the experiments described in Fig 6J were selected and shown.
